# Epidemiology and aetiology of male and female sexual dysfunctions related to pelvic ring injuries: a systematic review

**DOI:** 10.1007/s00264-021-05153-8

**Published:** 2021-08-10

**Authors:** Giuseppe Rovere, Andrea Perna, Luigi Meccariello, Domenico De Mauro, Alessandro Smimmo, Luca Proietti, Francesco Falez, Giulio Maccauro, Francesco Liuzza

**Affiliations:** 1 Department of Orthopaedics and Traumatology, Fondazione Policlinico Universitario A. Gemelli IRCCS - Università Cattolica del Sacro Cuore, Largo Agostino Gemelli, 8, 00168 Rome, Italy; 2 Department of Orthopaedics and Traumatology, AORN San Pio, Benevento, Italy; 3 Santo Spirito in Sassia Hospital, 00193 Rome, Italy

**Keywords:** Sexual disfunction, Pelvic ring injuries, Penile erection, Dyspareunia, Lumbosacral plexus

## Abstract

**Introduction:**

Pelvic ring injuries, frequently caused by high energy trauma, are associated with high rates of morbidity and mortality (5–33%), often due to significant blood loss and disruption of the lumbosacral plexus, genitourinary system, and gastrointestinal system. The aim of the present study is to perform a systematic literature review on male and female sexual dysfunctions related to traumatic lesions of the pelvic ring.

**Methods:**

Scopus, Cochrane Library MEDLINE via PubMed, and Embase were searched using the keywords: “Pelvic fracture,” “Pelvic Ring Fracture,” “Pelvic Ring Trauma,” “Pelvic Ring injury,” “Sexual dysfunction,” “Erectile dysfunction,” “dyspareunia,” and their MeSH terms in any possible combination. The following questions were formulated according to the PICO (population (P), intervention (I), comparison (C), and outcome (O)) scheme: Do patients suffering from pelvic fracture (P) report worse clinical outcomes (C), in terms of sexual function (O), when urological injury occurs (I)? Is the sexual function (O) influenced by the type of fracture (I)?

**Results:**

After screening 268 articles by title and abstract, 77 were considered eligible for the full-text analysis. Finally 17 studies that met inclusion criteria were included in the review. Overall, 1364 patients (902 males and 462 females, M/F ratio: 1.9) suffering from pelvic fractures were collected.

**Discussion:**

Pelvic fractures represent challenging entities, often concomitant with systemic injuries and subsequent morbidity. Anatomical consideration, etiology, correlation between sexual dysfunction and genitourinary lesions, or pelvic fracture type were investigated.

**Conclusion:**

There are evidences in the literature that the gravity and frequency of SD are related with the pelvic ring fracture type. In fact, patients with APC, VS (according Young-Burgess), or C (according Tile) fracture pattern reported higher incidence and gravity of SD. Only a week association could be found between GUI and incidence and gravity of SD, and relationship between surgical treatment and SD. Electrophysiological tests should be routinely used in patient suffering from SD after pelvic ring injuries.

## Introduction

Pelvic ring injuries, frequently caused by high-energy trauma, are associated with high rates of morbidity and mortality (5–33%), often due to significant blood loss and disruption of the lumbosacral plexus, genitourinary system, and gastrointestinal system [1–4].

Genitourinary injuries (GUI) and sexual dysfunctions (SD) associated to pelvic ring disruption are the result of direct or indirect trauma [5, 6]. Urogenital system structural and functional damages could be related to the anatomical relationship between the abdominal organs, neuro-vascular structures, and the pelvic ring [3]. Sexual dysfunctions represent an underestimated consequence of pelvic injuries mostly in young and sexually active patients, often cause of depression and quality of life reduction [2, 3].

Some authors observed a direct correlation between the Injury Severity Score (ISS) increase and the sexual disturbance incidence [7]. Some factor such as age, pelvic fracture complexity, and pubic symphysis alterations could represent erectile dysfunction risk factors after major pelvic injuries. Moreover elderly patients seem to be prone to experience impotence and SD after a pelvic ring trauma respect to younger patients [8, 9]. On the other hand, urethral trauma, pelvic organ prolapse, and urinary impairment are most frequent in women, as a consequence of posterior pelvic fractures [3, 5]. A direct trauma on the pelvic floor or pelvic soft tissue damage (such as connective tissue, neuro-vascular structures) could lead to pelvic floor dysfunction, urogenital and neurogenic pain, and dyspareunia [10].

Some literature review were performed on this topic, however, reported data were fragmentary and non-conclusive; furthermore, there is no consensus in the literature about the relationship between the type of pelvic fracture, the treatment received, and the urological/gynecological lesions and sexual dysfunctions in male and female patients. The aim of the present study was to perform a systematic literature review on male and female sexual dysfunctions related to traumatic lesions of the pelvic ring and to verify the possible association between the type of pelvic ring injury, the received treatment, and the gravity of sexual dysfunction.

## Material and methods

### Study setting and design

**Fig. 1:**
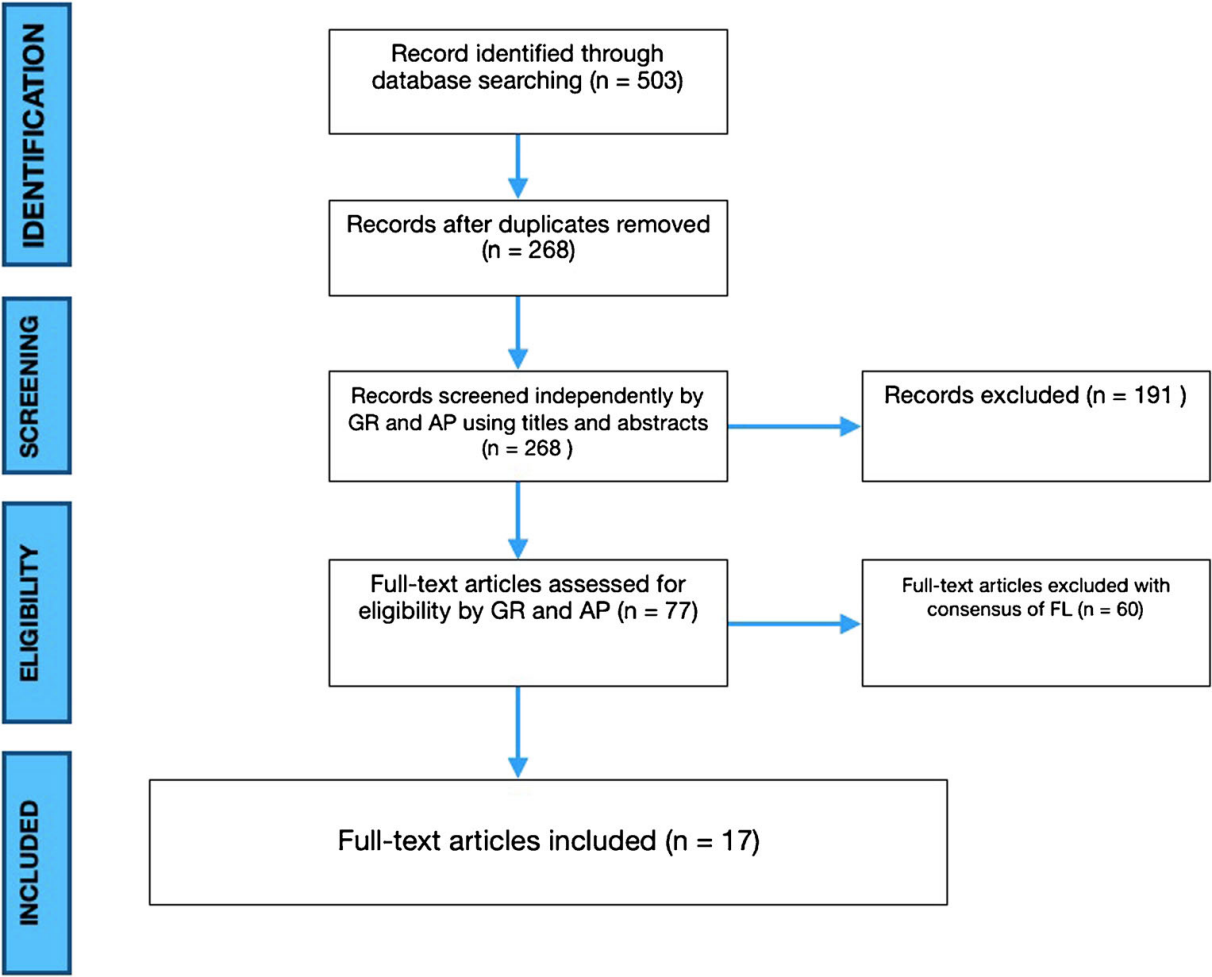
PRISMA flowchart

The present investigation represents a systematic literature review reported according to the Preferred Reporting Items for Systematic Reviews and Meta-Analyses (PRISMA) guidelines (Figure 1) [11].

### Review questions

The review questions were formulated following the PICO scheme (population (P), intervention (I), comparison (C), and outcome (O)) as follows:

- Do patients suffering from pelvic fracture (P) report worse clinical outcomes (C), in terms of sexual function (O), when urological injury occurs (I)?
- Is the sexual function (O) influenced by the type of fracture (I)?

### Inclusion and exclusion criteria

In this review, we considered the studies published as full-text articles in indexed journals, which investigated the association between pelvic ring injuries and sexual dysfunction. Only articles written in English with available abstract were included. No publication date limits were set. Surgical technique reports, expert opinions, case report, letter to the editor, studies on animals, unpublished reports, cadaver or in vitro investigations, review of the literature, abstracts from scientific meetings, and book chapters were excluded from the present review.

### Search strategy and study selection

Scopus, Cochrane Library MEDLINE via PubMed, and Embase were searched using the keywords: “Pelvic fracture,” “Pelvic Ring Fracture,” “Pelvic Ring Trauma,” “Pelvic Ring injury,” “Sexual dysfunction,” “Erectile dysfunction,” “dyspareunia,” and their MeSH terms in any possible combination. The reference lists of relevant studies were screened to identify other studies of interest. The search was reiterated until December 15, 2020.

### Data extraction

Two independent reviewers (A.P. and G.R.) collected the data from the included studies. Any discordances were solved by consensus with a third author (F.L.). For each study included in the present analysis, the following data were extracted: demographic features, type of fracture, traumatic mechanism, presence of risks factors, presence of associated urethral or bladder injuries, presence of other associated injuries, presence of dyspareunia (in females patients) or erectile dysfunction, treatment performed, possible complications and outcomes, hospital stay, and follow-up.

### Primary and secondary outcome measures

The primary outcome was the correlation between the type of fracture and the incidence of SD. The secondary outcomes were represented by the correlation between the treatment received and the incidence of SD.

### Statistical analysis

**Fig. 2:**
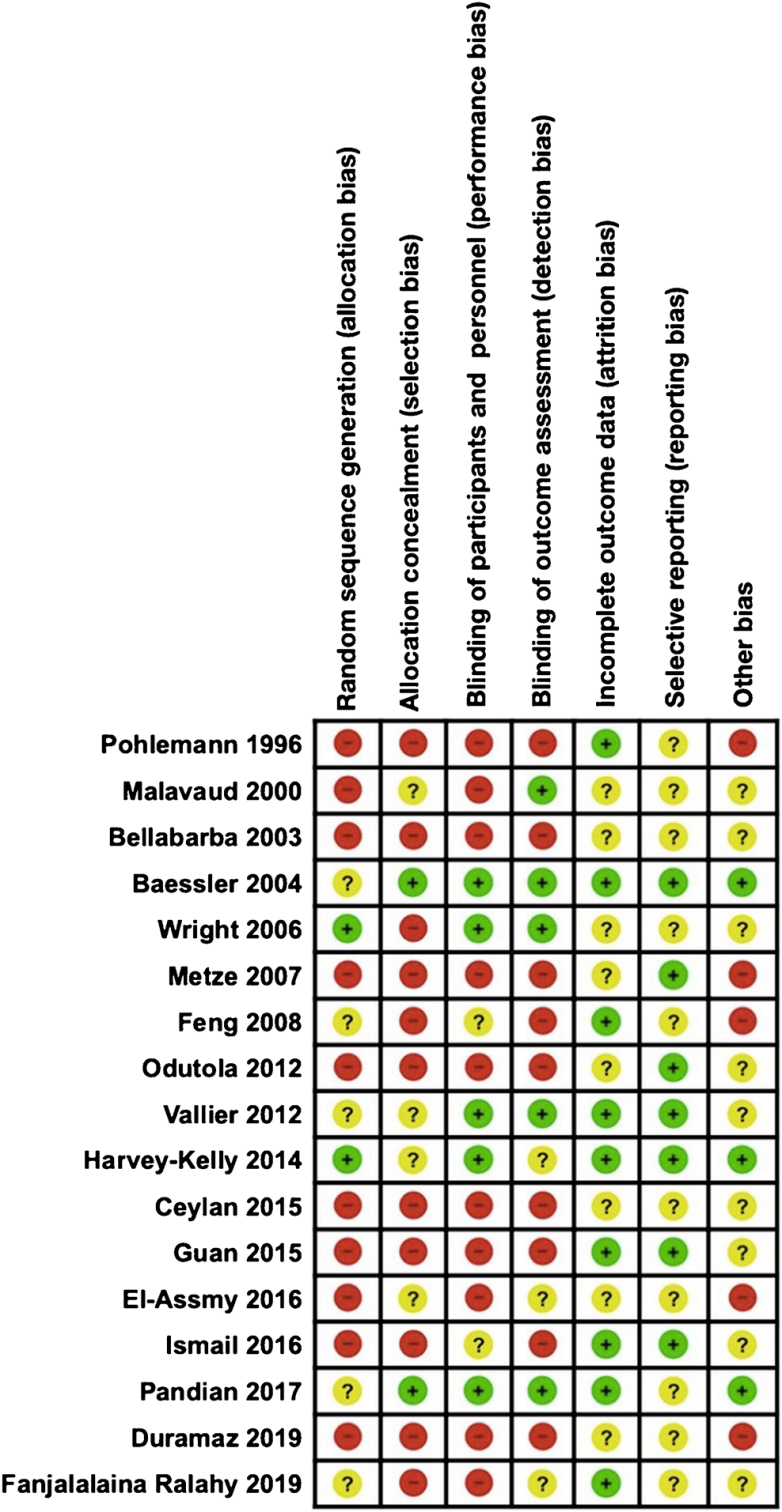
Risk of bias assessments of included studies using Cochrane risk of bias tool. Risk of bias is judged in each domain of selection bias, performance bias, detection bias, attrition bias, reporting bias, and other bias. Green: domain judged to be at low risk of bias; yellow: domain judged to be at unclear risk of bias; red: domain judged to be at high risk of bias

Risk of bias assessments and quality assessment of included studies was checked using Cochrane risk of bias tool (Figure 2). Numbers software (Apple Inc., Cupertino, CA) was used to tabulate the obtained data. Categorical variables are presented as frequency and percentages. Continuous variables are presented as means and standard deviation. Only one decimal digit was reported and was rounded up.

## Results

### Study selection

After screening 268 articles by title and abstract, 77 were considered eligible for the full-text analysis. Sixty studies were excluded because they did not fulfill inclusion criteria. Finally, 17 studies that met inclusion criteria were included in the review [5, 10, 12–26] (Figure 1). Among them, one had a level of evidence II, four had a level of evidence III, while 11 had a level of evidence IV [27].

### Patient characteristics

Overall, 1364 patients (902 males and 462 females, M/F ratio: 1.9) suffering from pelvic fractures were collected. The mean age ± standard error was 35.6 ±1.71 years. The mechanisms of injury were reported in 1005 of 1364 patients (73.6%); the most common was high energy traffic accident in 876 cases (87.2%), high falling injury in 87 cases (8.6%) and other causes in 42 patients (4.2%). Mean follow-up was reported in 14 out of 17 studies (82.3%), and it was about 32.9 months. Five hundred seventy six (42.2%) of all patients were polytraumas with associated lesions. The most common associated injuries were as follows: urogenital (336 cases), musculoskeletal (277 cases), visceral (124), and head (72 cases) injuries. In 13 studies, for a total of 740 patients (54.2%), the pelvic fractures were classified according to the Tile classification (type A: 260 pt, type B: 266 pt, type C: 214 pt), while in three studies, for a total of 326 patients (23.7%), the Young-Burgess classification was used (Anterior and Posterior Compression type, APC: 94 pt; Lateral Compression type, LC: 133 pt; Vertical Shear type, VS: 89 pt; Composite Stress type: 10 pt). In one study, for a total of 298 patients (21.8%), the pelvic fracture classification was not reported. Demographic characteristics of the included studies are reported in Table 1.

**Table 1:** Clinical characteristic, fracture classification, treatment received (orthopedics and urologic), and associated complications of included patients

**Studies**	**Number of patients**	**Sex**		**Age (year)**	**Traumatic mechanism**			**Classification AO/Tile**			**Classification Y/B**				**Polytrauma (*n*** **of patients)**	**Associated lesions**				**Orthopaedic treatment**			**Urologic treatment**			**Complication**				
		**M**	**F**		**Traffic accident**	**Fall from height**	**Other**	**Tile A**	**Tile B**	**Tile C**	**APC**	**LC**	**VS**	**CM**		**Visceral**	**Muscoloskeletal**	**Head**	**Urogenital**	**Conservative**	**External fixation**	**ORIF/CRIF**	**Chronic catheter**	**Reparation of bladder or urethral tear**	**Urethral realignment**	**Infection**	**Pain**	**Non-union/malunion**	**TVP/TEP**	**Neurological**
**Pohlemann 1996**	58	40	18	_	58	_	_	_	28	30	_	_	_	_	_	_	_	_	6	_	3	55	_	_	_	2	13	6	10	21
**Malavaud 2000**	46	46	_	39.6 (±15.9)	_	_	_	27	5	14	_	_	_	_	33	_	29	_	4	_	_	_	_	_	-	_	_	_	_	_
**Bellabarba 2003**	10	7	3	36 (23–64)	10	_	_	_	10	_	_	_	_	_	7	2	8	2	1	1	1	8	1	6	_	_	_	_	_	_
**Baessler 2004**	24	_	24	24 (11–92)	16	6	_	_	16	8	_	_	_	_	4	_	_	_	4	6	4	14	_	_	_	_	13	_	_	_
**Wright 2006**	298	192	106	33.1 (18–59)	298	_	_	_	_	_	_	_	_	_	_	_	_	_	_	_	_	_	_	_	_	_	_	_	_	_
**Metze 2007**	77	77	_	35 (22–48)	44	33	_	27	22	28	_	_	_	_	61	43	75	36	1	_	_	27	_	1	_		19	_	_	_
**Feng 2008**	40	40	_	35.4 (18–54)	_	_	_	27	13	_	_	_	_	_	_	_	_	_	40	_	_	_	_	_	11	_	_	_	_	_
**Odutola 2012**	151	111	40	40 (16–76)	_	_	_	_	_	_	46	56	49	_	32	_	32	_	29	_	21	130	_	_	_	_	_	_	_	
**Vallier 2012**	187		187	33 (16–55)	162	16	9	_	101	86	_	_	_	_	28	23	28	_	18	107	16	58	_	15	_	3	31	1	_	
**Harvey-Kelly 2014**	80	48	32	44.1 (19–65)	50	18	12	_	_	_	23	40	5	_	66	34	73	23	11	42	_	38	_	_	_	_	2	12	_	_
**Ceylan 2015**	26	26	_	42.6 (20–68)	26	_	_	16	5	5	_	_	_	_	11	2	9	_	_	22	3	1	_	_	_	_	_	_	_	_
**Guan 2015**	120	120	_	37.6 (±6.3)	_	_	_	73	35	12	_	_	_	_	_	_	_	_	115	_	_	_	_	_	115	_	_	_	_	_
**El-Assmy 2016**	58	58	_	31.6 (±12.2)	58	_	_	45	5	8	_	_	_	_	_	_	_	_	50	_	_	_	_	_	58	_	_	_	_	_
**Ismail 2016**	26	17	9	30.54 (±10.8)	26	_	_	_	15	11	_	_	_	_	9	3	4	3	18	_	_	26	_	_	_	5	9	_	_	3
**Pandian 2017**	20	20	_	34 (17–61)	18	_	2	11	2	7	_	_	_	_	_	_	_	_	20	19	1	_	_	_	20	_	_	_	_	_
**Duramaz 2019**	95	52	43	34(18–50)	71	14	10	_	_	_	25	35	35	10	26	7	14	5	12	-	_	95	_	_	_	6	_	4	_	4
**Fanjalalaina Ralahy 2019**	48	48	_	39 (18–82)	39	_	9	34	9	5	_	_	_	_	11	10	5	3	7	46	2	_	_	_	_	_	_	_	_	
**Total**	1364	902	462	35.6 (± 4.9)	876	87	42	260	266	214	94	131	89	10	288	124	277	72	336	243	51	452	1	22	204	16	87	23	10	28

### Sexual dysfunction

Sexual dysfunctions (SD) represent a common complication of pelvic injuries. Among the analyzed patients, 525 (39.8%) reported SD. The most common conditions described were as follows: erectile dysfunction (ED) in 366 cases, male orgasmic dysfunction (MOD) in 39 cases, and dyspareunia (DYS) in 120 cases. ED represents a condition of inability to obtain or maintain a penile erection sufficient to perform a satisfying sexual intercourse [28]. MOD includes many disorders ranging from premature ejaculation to complete inability to ejaculate including retrograde ejaculation [28]. DYS in woman represents painful sexual intercourse [29]. In nine studies, the authors used the International Index of Erectile Function (IIEF) questionnaire for the evaluation of sexual dysfunction [13, 16, 20–22, 24–26]. Ad hoc questionnaire, Arizona Sexual Experiences Scale (ASEX) [25], Female Sexual Function Index (FSFI) [25], and Majeed and Hannover questionnaire were also used in other studies [23]. In eight studies for a total of 291 patients, the gravity of SD was reported. In 78 patients, SD was classified as severe, in 61 as moderate, in 116 as mild to moderate, and in 36 as mild (Table 2).

**Table 2:** Epidemiology and evaluation of sexual dysfunction after pelvic ring injury

**Studies**	**Number of patients**	**Sexual dysfunction**				**Evaluation scale**			**Dysfunction quantification**			
		**Erectile dysfunction**	**Ejaculation disturbances**	**Dyspareunia**	**Percentage**	**IIEF**	**ASEX**	**Other**	**Mild**	**Mild to moderate**	**Moderate**	**Severe**
**Pohlemann 1996**	58	5		1	10.3%	_	_	Ad hoc questionnaire				
**Malavaud 2000**	46	11		_	23.9%	X	_	_	4	5	1	1
**Bellabarba 2003**	10	1	_	2	30.0%	_	_	Ad hoc questionnaire	_	_	_	_
**Baessler 2004**	24	_	_	8	30.0%	_	_	Golombok Rust Inventory of Sexual Satisfaction	_	_	_	_
**Wright 2006**	298	39		23	20.8%	_	_	FCI questionnaire		55		7
**Metze 2007**	77	16	_	_	19.5%	X	_	_	4	2	1	8
**Feng 2008**	40	11	_	_	27.5%	_	_	Nocturnal penile tumescence, Doppler ultrasonography	_	_	_	_
**Odutola 2012**	151	21	30	1	34.4%	_	_	Sexual functioning questionnaire	_	_	_	_
**Vallier 2012**	187	_	_	48	25.7%	_	_	_	_	_	_	_
**Harvey-Kelly 2014**	80	25	_	14	48.7%	X	_	FSFI, EQ5D	_	_	_	_
**Ceylan 2015**	26	26		_	100%	X				14		12
**Guan 2015**	120	115	_	_	95.8%	X	_	_	16	34	47	18
**El-Assmy 2016**	58	42	_	_	72%	X	_	_	6	6		19
**Ismail 2016**	26	10	_	_	38%	_	_	Majeed, Hannover	_			
**Pandian 2017**	20	15	_	_	75%	X	_	_	_	_	8	7
**Duramaz 2019**	95	13	9	23	47.4%	X	X	FSFI	_	_	_	_
**Fanjalalaina Ralahy 2019**	48	16	_	_	33.3%	X	_	_	6		4	6
**Total**	1364	366	39	120	39.3 (±24.6)%	9	1	9	36	116	61	78

## Discussion

Pelvic fractures represent challenging entities, often concomitant with systemic injuries and subsequent morbidity [30]. They are relatively uncommon (3–9% of all fractures), but their incidence increases up to 20% in polytraumatized patients [30]. Sexual dysfunctions after pelvic injuries are common but underestimated complications and are generally caused by high-energy trauma in young and usually sexually active subjects [1–6]. In fact, it has been shown that for a 1-point increase in Injury Severity Score, there is a 2% increase of sexual dysfunction [7]. The aim of this research is to provide a detailed analysis of the SD etiology after a pelvic injury and to examine possible factors that may increase the incidence and risk of SD in these patients.

### Anatomical consideration and classification systems

The pelvis is an osteo-ligamentous ring that connects the spine to the lower limbs allowing the weight-bearing force transmission [31]. Its stability depends on strong ligamentous structures such as the anterior symphyseal and the sacrospinous ligaments that oppose external rotation, the sacral-tuberous ligaments which resist shear and flexion, and the sacro-iliac ligament that confers stability on vertical plane [32]. The urethra crosses the urogenital diaphragm and the perineal membrane and is very close to the anterior arch of the pelvis. In particular, in males, the membranous urethra pierces the perineal membrane and is stabilized by the pubo-prostatic ligaments [31]. For this reason, especially pubic bone fractures or diastasis may cause urethral injuries. Considering this complex anatomy, high energy trauma of the pelvic ring could create mechanical and hemodynamic instability with loss of blood and disruption of the genitourinary system, the gastrointestinal system, and the lumbosacral plexus [1–6]. Different classification systems for pelvic injuries have been developed to identify different types of fractures based on the traumatic mechanism and associated injuries (Young and Burgess Classification) [33] or on the stability of the pelvic ring (Tile classification) [34]. A comprehensive modern classification is the AO/OTA [35].

### Neurogenic etiology

Most of the previous papers that studied the etiology of SD after pelvic injuries did not differentiate the neurogenic factors from the vascular ones [18–21]. The nervous supply damage to the penis or clitoris seems to be one of the most important factors. Erection depends on a combination of psychophysical stimuli, and it is controlled by parasympathetic system whose fibers originate from S2 to S4 nervous roots [21]. These nerves in fact, as part of the somatic nervous system, participate to the erectile mechanism by providing sensation to the skin of the penis [36]. A similar mechanism was involved in women and causes clitoris swelling and engorgement as well as vagina lubrication [37].

Differently to the erection, the predominant input for ejaculation was provided by sympathetic nervous system (T12–L2) which runs from the hypogastric plexus to the genital structure through the hypogastric nerve [37]. However, while the parasympathetic system is involved during reflexogenic penile erection (physical stimulation), sympathetic system could cause psychogenic penile erection [37]. Damage to the S2–4 nervous roots after ilium fractures and sacro-iliac diastasis could cause sexual or urination dysfunction by stretching the S2–4 nerve roots and their branches (pudendal nerve and dorsal nerve of the penis) with interruption of the local reflex arc [12, 36]. This occurrence causes the well-known lower motor neuron syndrome (LMNS) [37]. Patient with LMNS lesions after pelvic trauma usually fail to have penile erections in response to tactile stimulation. Nevertheless in these patients, a psychogenic erection (mediated by sympathetic pathways T10–T12) could occur [37]. As for women, few studies are available in literature. It appears that clitoral enlargement and vaginal lubrication may occur in response to direct stimulation when the sacral reflex is intact and after psychogenic stimulation in the case of LMNS [38].

Although neurological etiology is one of the most frequently described [21], sensitive and direct neurophysiologic tools for neurological assessment are still missing. Electrophysiological tests used to diagnose neurogenic SD are as follows: posterior tibial somatosensory nerve evoked potentials (PTSSEPs), pudendal nerve evoked potentials (PDEPs), and the bulbocavernosus reflex (BCR). However, these tests are often neglected or not routinely performed in patients with pelvic trauma. [21]

### Vascular aetiology

A deficit of penis blood supply seems to be one of the most probable vascular causes of erectile dysfunction. The internal pudendal artery represents the only vascular supply to the corpus cavernosum. This artery had two branches. The first, so-called internal pudendal artery across the Alcock’s canal. At this level, pelvic injuries could damage the internal pudendal artery [36].

Veno-occlusive etiology as results of mechanic damage is not clear; however, it is well known that the veno-occlusion that occurs during erection in the corpus cavernosum is caused by increased arterial blood flow and smooth penile muscle relaxation. Some authors in fact hypothesized that traumatic injuries could cause smooth muscle structural changes which leads to SD [32].

### Correlation between sexual dysfunction and genitourinary lesions

The incidence of associated genitourinary injury (GUI), including bladder disruptions, injury to the bladder neck, and urethral injuries with associated sexual dysfunctions, ranges from 6.5 to 30% [39–41]. Only 14 of 17 studies evaluated the association between SD and concomitant genitourinary lesions after a pelvic ring fracture. Among these, 8 studies (47%) [10, 13, 14, 18, 20, 22–24] reported that genitourinary injuries represent an additional risk factor for the development of SD, both in man and women. However, the correlation seems to be moderate and mostly not statistically significant. In 6 studies (35.3%), the authors assumed that the presence of a genitourinary lesion did not represent a risk factor for the development of SD after a fracture of the pelvic ring. In three studies (17.7%), the correlation between SD and genitourinary injuries was not analyzed.

### Correlation between sexual dysfunction and pelvic fracture type

The pelvic lesions most frequently associated with sexual dysfunctions are the ones that involve bilateral pubic rami, the symphysis diastasis, and fractures or dislocations of the sacroiliac joints. [9]. Many authors have described an association between sacro-iliac and pubic symphysis injuries and urogenital impairment in both sexes [10, 20, 25].

Sixteen of 17 studies evaluated the association between SD and the type of pelvic fracture. Regarding the Tile classification, the authors of five studies (29.4%) sustained that a pelvic fracture represents an independent risk factor for the development of SD; besides, there is not any statistically significant correlation between the Tile classification and the gravity or the frequency of SD. In another seven studies (41.2%), a statistically significant correlation was found between the complexity of the fracture and the frequency of SD. In these studies, Tile B and C fractures were associated with a higher incidence of SD in both males and females. For the Young-Burgess classification, a statistically significant correlation was found in all studies (4, 23.5%) between the presence of APC or VS fractures and the gravity and frequency of SD. Other factors that may influence the gravity and the incidence of SD after pelvic fracture with statistically significant results seem to be sacroiliac fractures, lumbar transverse process fractures, symphyseal diastasis (> 25 mm), and pubic rami fractures.

### Other correlations

A noteworthy correlation concerns the association between SD and surgical treatment in patients with pelvic fractures. The authors of two studies [10, 25] sustained that symphyseal plating was associated with higher incidence of DYS in their series. Harvey-Kelly et al. [20], instead, sustained that an ORIF of the pelvic fracture represents a higher risk for SD compared with CRIF techniques and/or conservative treatment.

### Treatment and complications of pelvic ring injuries

Pelvic ring fracture management is closely related to the pelvic ring stability, defined as the capacity to support physiologic load when the patient is in sit, lie, or stand position [42].

Isolated anterior pelvic ring fractures (type A) are usually stable and treated conservatively, while, injuries with posterior ring or combined anterior and posterior pelvic ring involvement (types B and C) are usually considered unstable and surgical stabilization should be considered [42]. Non-operative patients should be followed for at least one year to determine the outcome and maintenance of the reduction. Weight bearing is usually gradually allowed using crutches or a walker after a variable period of bed rest [42]

Surgical treatment is needed in all cases of instability and/or deformity of the pelvic ring and many techniques exist for both open and closed reductions, but the main issue remains achieving anatomic restoration of the pelvis [43].

Owing to the important wound complications following open surgery, less invasive techniques, such as percutaneous screw fixation, have been largely used over the last few years to stabilize the posterior pelvic ring and lumbo-sacral junction while reducing complications [44–50].

Among the analyzed studies, only 12 reported the orthopaedic treatment performed in 746 patients. In 452 of 746 cases (60.6%), open reduction internal fixation (ORIF) or closed reduction internal fixation (CRIF) was performed, 51 patients (6.8%) were treated with external fixation whereas 243 (32.6%) were treated conservatively with bed rest and physiotherapy (Table 1).

Anastomotic urethroplasty remains the gold standard for the treatment of pelvic fractures urethral injuries (PFUI) and the aim of this surgery is to allow a tension free bulbo-membranous anastomosis [51]. In seven studies for a total of 227 patients, an urological treatment was performed. In 204 of 227, a urethral realignment was performed while in 22 cases, a bladder or urethral reparation was performed.

Regarding neurosurgical repair of sacral plexus and peripheral genital nerves, or sacral electrical stimulation, some surgical procedures were proposed principally for patients with spinal cord injuries (SCI) [52]. The peripheral nerve transfer into the nerves/roots below the injury seems to be a promising approach. It could permit urinary and sexual function recovery in patients with complete or incomplete S2–S4 lesions; however, only a few cases were described [52]. Sacral anterior root stimulation (SARS) and later sacral deafferentation (SDAF) introduced by Brindley [53] primarily for neurogenic bladder dysfunction in SCI individuals could be an option for neurogenic sexual dysfunction management [54]. Zaer et al. in a recent study with 287 patients [54] described a compensation of erection reflex due to electrostimulation reflex erection in 30% of male patients after SARS-SDAF procedures. On the other hand, no significant differences were observed in female patients corroborating the theory that the women’s sexual cycle is more controlled by mental excitements than physical symptoms [54].

Excluding sexual dysfunction, seven of the 17 studies analyzed reported the complications related to the fracture or to the received treatment. In 87 patients, a persistent pain at the last follow-up was observed. In 28 cases, neurological complications were found. In 23 patients, a non-union or malunion was detected. In 16 patients, a superficial or deep wound infection was observed. Thromboembolic complications were described in ten patients (Table 1).

### Limitations

This study has some limitations. First, most of the studies included in the analysis were retrospective case series with no comparative group; unfortunately, no higher quality studies have been performed on the subject due to its high complexity; in fact, it is not possible to perform randomized clinical trials or double-blind controlled studies. Second, there is variability in age groups and also follow-up. Third, there is a lack of homogeneity in reporting fracture classification, evaluation scales, treatment, and outcomes.

## Conclusions

SD after pelvic ring injuries, in both male and female patients, represent a common consequence, with important influence on quality of life, especially in young patients.

There is evidence in the literature that the gravity and frequency of SD are related with the pelvic ring fracture type. In fact, patients with APC, VS (according Young-Burgess), or C (according Tile) fracture pattern reported higher incidence and gravity of SD. Only a week association could be found between GUI and incidence and gravity of SD, and relationship between surgical treatment and SD. Electrophysiological tests should be routinely used in patients suffering from SD after pelvic ring injuries. A multidisciplinary approach involving orthopedist, urologist, neurologist, and neurosurgeon should be recommended in the treatment of sexual dysfunction following pelvic injuries.

## Data Availability

Not applicable

## Declarations

All patients provided the informed consent for the publication of the clinical history. The study was authorized by the local ethical committee and was performed in accordance with the Ethical standards of the 1964 Declaration of Helsinki as revised in 2000.
